# Sexually Transmitted Infections in Adolescents and Young Adults: A Cross Section of Public Health

**DOI:** 10.3390/ijerph21040501

**Published:** 2024-04-19

**Authors:** Nunzia Cannovo, Elena Bianchini, Luciana Gironacci, Elisabetta Garbati, Filiberto Di Prospero, Mariano Cingolani, Roberto Scendoni, Piergiorgio Fedeli

**Affiliations:** 1 Legal Medicine Unit, Local Health Authority (AST) 3, 62032 Camerino, Italy; nunzia.cannovo@gmail.com; 2 Clinical Governance and Risk Unit, Macerata Hospital, Local Health Authority (AST) 3, 62100 Macerata, Italy; elena.bianchini@sanita.marche.it; 3 Analysis Laboratory Unit, Local Health Authority (AST) 3, 62012 Civitanova Marche, Italy; luciana.gironacci@sanita.marche.it; 4 Gynecology and Obstetrics Unit, Civitanova Marche Hospital, Local Health Authority (AST) 3, 62012 Civitanova Marche, Italy; elisabetta.garbati@sanita.marche.it (E.G.); filiberto.diprospero@sanita.marche.it (F.D.P.); 5 Department of Law, Institute of Legal Medicine, University of Macerata, 62100 Macerata, Italy; mariano.cingolani@unimc.it; 6 School of Law, Legal Medicine, University of Camerino, 62032 Camerino, Italy; piergiorgio.fedeli@unicam.it

**Keywords:** sexually transmitted infections, sexual contact, adolescents, young adults, vaginal sex, anal sex, oral sex, infectious diseases

## Abstract

Introduction. Sexually transmitted infections (STIs) can be caused by a number of microorganisms that vary greatly in size, life cycle, clinical manifestations, and sensitivity to available treatments. Transmission of STIs can occur during unprotected (or condomless) sexual contact and through the exchange of body fluids during any type of activity. The prevalence of sexually transmitted diseases remains high in the world, despite diagnostic and therapeutic improvements for these infectious diseases that rapidly eliminate the contagiousness of patients. Our study determines the prevalence of STI pathogens in adolescents and young adults in the population of the Province of Macerata (Italy). We will analyze data in correspondence to age and gender, and we will compare our results to international studies. Materials and Method. We analyzed STI test results from the entire database of a Provincial Health Authority for the period 2021–2022. The samples came from the following age groups: 0–12, 13–18, 19–25, and 26–35 from 2021 to 2022. The results came from vaginal and cervical swabs (for females); urethral, rectal, and pharyngeal swabs (for males and females); and seminal fluid (for males) for the following infections: HPV, *Chlamydia trachomatis*, *Mycoplasma genitalium*, *Ureaplasmas*, *Gardnerella*, *Trichomonas vaginalis*, *Neisseria gonorrhoeae*, and *Treponema pallidum*. The results also came from blood tests for HIV, hepatitis C, hepatitis B, and *Treponema pallidum* (TPHA, VDRL). In addition, we examined results from urine tests for chlamydia, *Neisseria gonorrhoeae*, trichomonas, and *Treponema pallidum*. Conclusions. The literature for other countries reports the need for comprehensive, culturally and developmentally sensitive care to address sexuality-related issues in adolescents and young adults, a need that also applies to Italy. These data will be of great importance in adopting evidence-based STI control programs in Marche Region. This study could, indeed, represent a landmark for public health officials and professionals, with the aim of promoting adolescents’ access to sexual health services to receive useful information, strengthening preventive measures in younger age groups, and designing sexual education programs.

## 1. Introduction

The term sexually transmitted infections (STIs) encompasses a variety of clinical syndromes caused by pathogens prevalently acquired and transmitted through sexual activity [1] conducted through vaginal sex, anal sex, oral sex, and using fingers, other body parts (frottage or skin-to-skin rubbing/contact), or sex toys that have come in contact with another person’s genitals or body fluids [2].

The WHO [3] estimates that every year in the world there are 357 million new cases of sexually transmitted infections in women and men between the ages of 15 and 49. The European Center for Disease Prevention and Control (ECDC) (4) reports that STIs are the second most widespread type of infection, after respiratory infections, and similar findings have been reported for the United States as well [4,5].

The prevalence of STIs varies from region to region and according to sex, but it has a significant impact on the health and life of children, adolescents, and adults throughout the world [6].

In Italy, from 1991 to 2017, the most frequent STIs were ano-genital condylomas (54,272 cases), latent syphilis (10,736 cases), genital herpes (9409 cases) and nongonococcal non-chlamydial bacterial cervicovaginitis (Ng-Nc), that is, from different etiological agents than *Chlamydia trachomatis* (Ct), *Neisseria gonorrhoeae* (Ng), and *Trichomonas vaginalis* (Tv) (9108 cases, 7.1% of the total) [7], data which may underestimate the reality due to an ineffective notification system [8].

The factors [9] that limit the control of sexually transmitted infections include:

- Unprotected and condomless sex with promiscuity (increased number of relationships);
- Cultural reticence about speaking of sexual topics [10];
- Inadequate funding for prevention efforts, diagnostic tests, and existing treatments and for the development of new tests and treatment;
- Predisposition to re-infection when both partners are not treated contemporaneously;
- Incomplete treatment, which can lead the microorganisms to develop drug resistance;
- International travel, which facilitates rapid global spread of STIs.

The prevalence of STIs in the world remains high, [11] notwithstanding diagnostic and therapeutic improvements that rapidly eliminate the contagiousness of patients affected by most of these infections.

The adolescent population is also affected by the spread of STIs. The behavioral factors associated with the acquisition of STIs in adolescents include [12,13,14,15,16,17,18]:

- time passed since first sexual intercourse;
- sexual activity in early and mid-adolescence;
- multiple partners, new partners, or partners who have several other partners;
- inconsistent use of condoms, especially with regular partners;
- alcohol and drug use;
- rectal douching or enemas in preparation for receptive anal sex.

The control of STIs is one of the priorities of the WHO [7], whose strategy includes identification and reporting to healthcare authorities as well as prevention through educational programs and informational/formative moments to teach sexually responsible behavior [19,20,21].

These infections are caused by bacteria, viruses, protozoa, and parasites as widely reported in the specialist literature [22,23,24,25,26,27]. If not diagnosed and correctly treated, STIs can cause significant harm to the male and female reproductive systems [28].

The clinical presentation of STIs varies in relation to the type of infection [29,30]. In general, STIs such as gonorrhea or chlamydia cause inflammation [31], while others such as herpes simplex, syphilis, or chancroid cause ulceration [32], which in turn predisposes patients to other infections such as HIV or in some cases, to neoplastic lesions [33]. Untreated STIs can also have serious systemic health complications (e.g., neurosyphilis, HIV-AIDS, disseminated gonococcal disease, to name a few), as well as perinatal/pregnancy complications. Really, many STIs can be completely asymptomatic or subclinical, thus the patient is unaware of infection.

For the diagnosis of many infections, there are laboratory exams [34] on blood or on liquids on the genital or rectal mucus. At times, the microorganism responsible cannot be identified and the diagnosis is based on clinical information. For this reason, the literature stresses that gynecological checkups should begin in adolescence [35].

Normal psychosocial development in adolescents includes the desire for autonomy and the increase of risky behavior, which makes adolescents particularly vulnerable to STIs, as reported in the literature [27]. Thus, we thought it would be useful to ascertain whether a similar tendency holds in a local Italian reality.

Our retrospective study examined and compared the prevalence of STIs between the years 2021–2022 stratified by patient age, pathogen type, and geographic region (Province of Macerata, Italy). The idea for this work was born following the Marche Region’s participation in the European Testing Week [36], a week of extraordinary mobilization which has as its objective the promotion of tests for HIV, viral hepatitis, and sexually transmitted infections and the promotion of awareness of the benefits of an early diagnosis of these infectious diseases.

Furthermore, the Regional Prevention Plan 2020–2025 (approved with DGR 1640/2021) [37] has provided for an “Infectious Diseases and Vaccinations” program dedicated to the prevention of STIs and a denominator “Schools that promote health”, aimed at offering development subsidies and support actions aimed at improving the well-being of all those who, in various capacities, move within the school context, as well as promoting conscious behavior to maintain their health, therefore, also offering information on STIs.

The authors therefore felt the need to verify how widespread the STIs were by taking the Province of Macerata as a regional sample, which consists of more than 300,000 inhabitants, representing approximately 20% [38] of the population resident in the Marche Region, and then comparing these data with the international results and verifying whether there is an STI emergency in the sample examined, whether the data are in line with the literature, and whether there is a need for targeted information.

In fact, the choice to carry out subgroups arises from the need to verify whether it is necessary to identify a target of people who need greater attention from the regional health system in terms of information campaigns.

Since all the Provinces of the Marche Region (Ancona, Macerata, Pesaro-Urbino, Ascoli Piceno) are made up of three geomorphological areas—mountainous area, hilly area, and maritime area—the authors carried out the study respecting these characteristics in order to verify whether they reflected changes in the prevalence of the STIs, it seems with a view to suggest possible differentiated information campaigns.

## 2. Materials and Methods

We conducted a retrospective study. We analyzed STI test results from the period 2021–2022 from the entire database of a provincial public hospital (Macerata) and of three public hospitals belonging to the same Province (Civitanova Marche, Camerino, and San Severino Marche), which serve a population of 30,486 individuals.

### 2.1. The Setting

The samples came from the following age groups: 0–12, 13–18, 19–25, and 26–35; the first group serves as a target, because it is assumed that members of this group do not yet have sexual activity.

The Province of Macerata is located in the Marche Region and groups together 57 municipalities; its territory is commonly distinguished by its geomorphological characteristics into a mountainous area (Camerino and surrounding areas), a hilly area (Macerata and surrounding areas), and a maritime area (Civitanova Marche and surrounding areas).

The population has a prevalence of inhabitants over the age of 65 with variability between the three identified areas; in fact, the old age index of the Province of Macerata as a whole is 212.1 (as of 1/1/22), while that of Camerino is 304.6 (mountain area), that of Macerata is 226.5 (hilly area) and that of Civitanova is 188 (maritime area).

It is clear that there is a prevalence of the elderly population more concentrated in the mountainous and hilly areas of the Province of Macerata, this determines socio-economic differences in favor of the hilly and maritime areas [39].

The choice to examine the data also in terms of the mountainous, hilly, or coastal geographic zones was motivated by the awareness that these zones provide different levels of healthcare services, and consequently an interest in verifying whether this may be a deterrent to being tested for STIs. In addition, given that the coastal areas are marked by a significant phenomenon of female prostitution, we wanted to look at the data on related target infections (viral hepatitis, HIV, syphilis, gonorrhea, chlamydial infection, and trichomoniasis).

The age groups were chosen on the basis of practical criteria. The 0–12 group, not sexually active, was included in the study as a target group; infections would be expected to be transmitted vertically or through contamination in communities with unhygienic conditions. The age of 13 in the 13–18 group was chosen in reference to the penal code article 609 quarter, which decriminalized sexual acts on minors over the age of 13. Also, this age group covers the years of adolescence. It was thought that individuals in the other two age groups, 19–25 and 26–35, would be sexually mature and have some notions about STIs.

### 2.2. Study Design

We considered only the tests conducted for subjects for whom date of birth and sex were known. Anonymized tests for which only the sex was known were excluded, as were samples that provided insufficient information or were poorly conserved because the samples had been collected poorly. The number of exams excluded is 0.81% of the total of those carried out.

Personal data collection was performed by accessing the archive program (Alchimia 2002) created for clinical purposes, and the data on all the STI laboratory tests conducted for the public Hospitals of Macerata, Camerino, Civitanova Marche, and San Severino Marche. 

The literature on STIs [4] focuses on *Chlamydia trachomatis*, *Neisseria gonorrhoeae*, Mycoplasma genitalium, *Trichomonas vaginalis*, HIV, HPV, and viral hepatitis (hepatitis C, hepatitis B).

Our study excluded protozoa and parasites due to poor response, but expanded the list of bacterial and viral infections to include HPV, *Chlamydia trachomatis*, Mycoplasma genitalium, Ureaplasmas, Gardnerella, *Trichomonas vaginalis*, *Neisseria gonorrhoeae*, *Treponema pallidum*, *Trichomonas vaginalis*, HIV, hepatitis C, and hepatitis B. 

#### STI Panel Collection

Specimens consisted of vaginal/endocervical swabs (for females), uretra (from men), rectal and pharyngeal swabs (for males and females), and seminal fluid (for males) for the following infections [40]: HPV, Chlamydia trachomatis, Mycoplasma genitalium, Ureaplasmas, Gardnerella, *Trichomonas vaginalis*, *Neisseria gonorrhoeae*, *Treponema pallidum*. We also analyzed results from blood tests for HIV, hepatitis C, hepatitis B, and *Treponema pallidum* (TPHA, VDRL), as well as results from urine tests for chlamydia, *Neisseria gonorrhoeae*, Trichomonas, and *Treponema pallidum*. 

### 2.3. Laboratory Method

In the Table 1, we report the laboratory methods used for each individual test performed.

### 2.4. Ethics Approval and Privacy Aspects

The data extraction was carried out by staff who work at the centralized laboratory of the Macerata Hospital, who have username and password and institutional authorization to access the data banks for management purposes. During the initial phase, the data extracted were anonymized, then input in anonymous form into a password-protected Microsoft Excel spreadsheet, which will be destroyed a year after the completion of this study. In respect of the principle of minimization, only the data needed for and pertinent to the research goals were examined. Only the demographic data on age and sex at birth in relation to the tests conducted were used, as they were essential for achieving the goals of the study. Thus, from the very beginning, the data collection excluded data with any references that could make it possible to arrive at the identity of individuals. 

In addition, the laboratory staff involved in the study followed the technical and organizational measures specified in the hospital data protection policy, in order to reduce to an acceptable level the risk of concrete threats to the rights and fundamental freedoms related to the treatment of personal data for the present study.

For various reasons, it was not possible to contact the individuals for whom the laboratory tests were conducted, or their legal representatives. First, they might have moved since the test was conducted. Second, the present study was financed by a not-for-profit entity, and lacked the funds and resources for such an effort. Third, the goal of the study was to have current data on the post-pandemic period, and the time necessary to contact the 9239 individuals in the databases would have caused an overlong delay in the completion of the study. 

The study methods complied with multiple EU and Italian regulations. The treatment of personal data for scientific research purposes was conducted in line with EU Regulation 2016/679 [41], the Italian Personal Privacy Code [42], the General Regulation on Data Protection [43,44] and the Provisions regarding treatment of personal data for goals of scientific research, attachments 4 and 5 of the measure of 5 June 2019, as well as the Deontological Rules [45] on treatment for statistical purposes or scientific research, which constitute the essential condition of legitimacy and correctness of the treatment (art. 2-quater of the Code and art. 21, Section 5 of the legislative decree of 10 August 2018, n. 101).

## 3. Results

The first datum available was the number of subjects who had tests performed, grouped by sex, age, and year (Table 2).

Then, we counted all the diagnostic checks performed on patients in the reference period, divided by sex and year (Table 3). In fact, each person underwent more than one test.

Next, observing that females had a higher number of tests performed than males, we noted that culture exams on vaginal swabs entail 15 tests for each swab sample.

**Figure 1:**
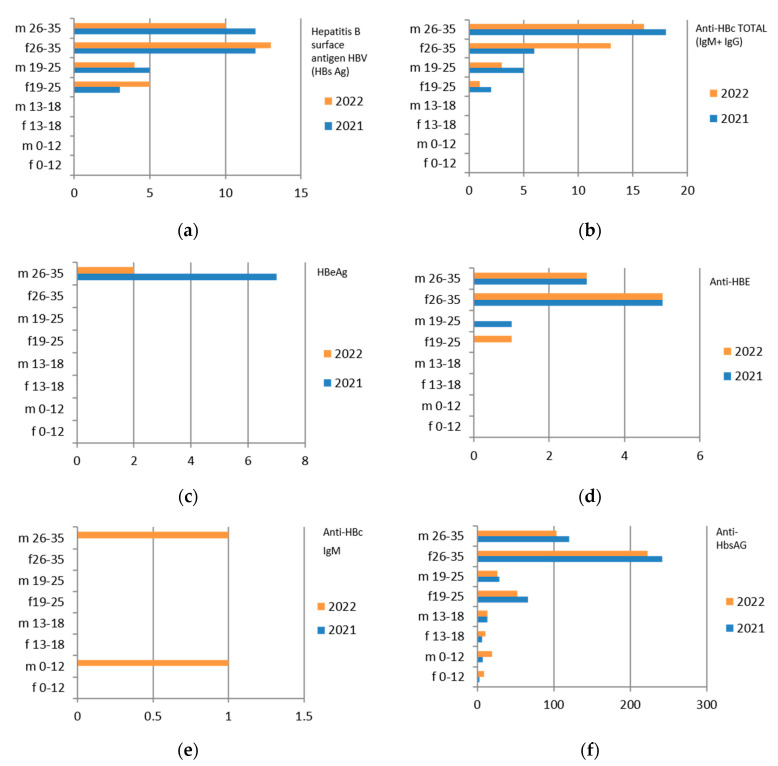
Estimated prevalence of HBV ((**a**) HBs Ag, (**b**) ANTI–HBc Ig Total, (**c**) HBeAg, (**d**) ANTI–HBE, (**e**) ANTI–HBc IgM, (**f**) ANTI–HbsAG) subdividing the data by sex, age group, and year (2021 and 2022).

**Figure 2:**
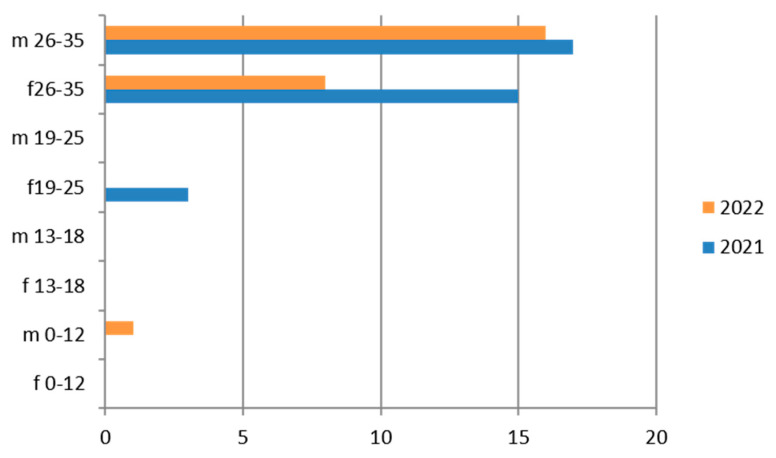
Estimated prevalence of HCV (Anti–HCV IgG), subdividing the data by sex, age group and year (2021 and 2022).

**Figure 3:**
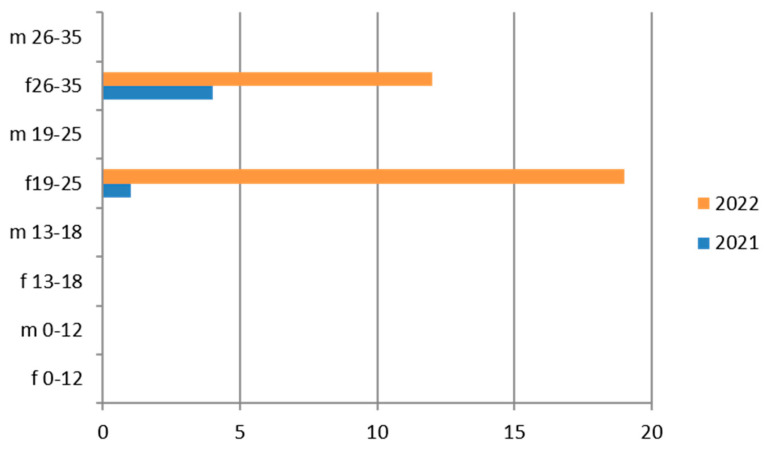
Estimated prevalence of *Chlamydia trachomatis* (*Chlamydia trachomatis* cervical swab), subdividing the data by age group and year (2021 and 2022).

**Figure 4:**
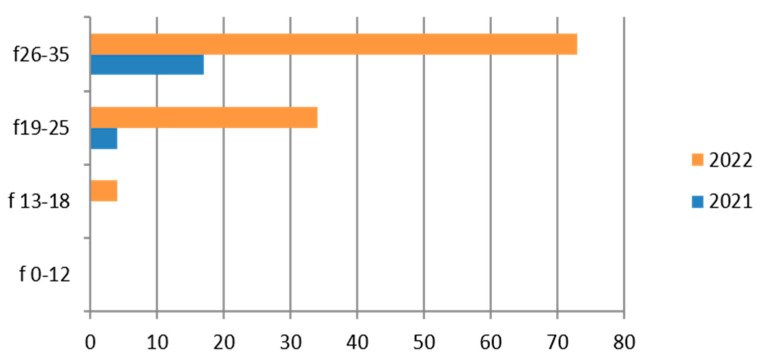
Estimated prevalence of *Gardnerella vaginalis* on vaginal swab, subdividing the data by age group and year (2021 and 2022).

**Figure 5:**
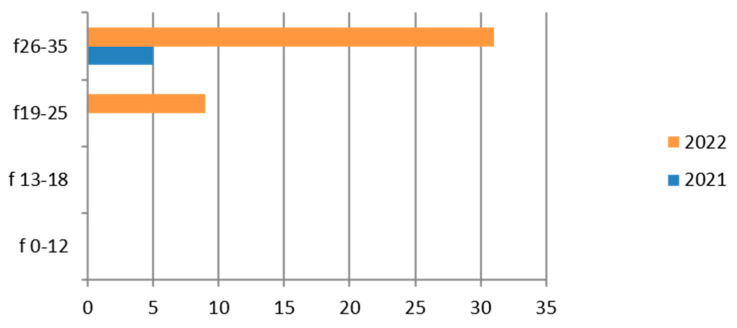
Estimated prevalence of HPV (HPV typing cervical swab), subdividing the data by age group and year (2021 and 2022).

**Figure 6:**
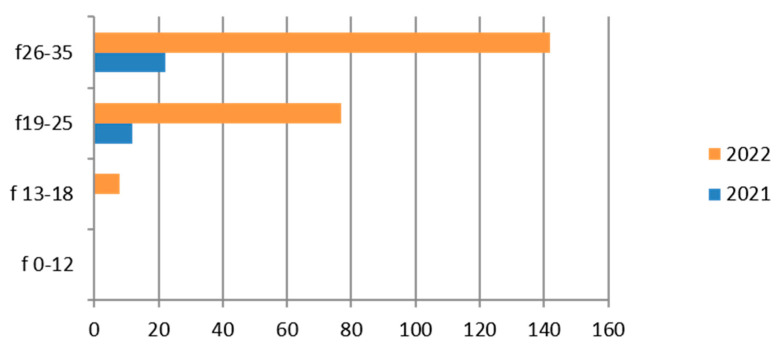
Estimated prevalence of Mycoplasma/Ureaplasma (vaginal culture), subdividing the data by age group and year (2021 and 2022).

**Figure 7:**
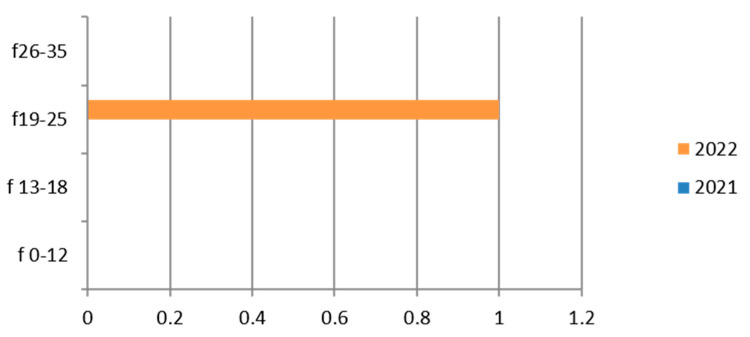
Estimated prevalence of Neisseria (*Neisseria gonorrhoeae* search cervical culture swab), subdividing the data by age group and year (2021 and 2022).

**Figure 8:**
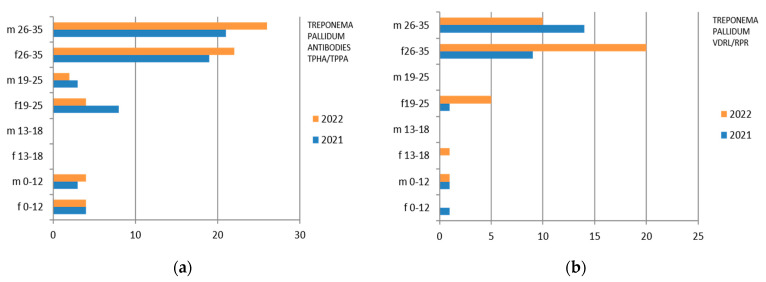
Estimated prevalence of Treponema Pallidum (**a**) Treponema antibodies TPHA/TPPA and (**b**) Treponema Pallidum VDRL/RPR), subdividing the data by sex, age group, and year (2021 and 2022).

**Figure 9:**
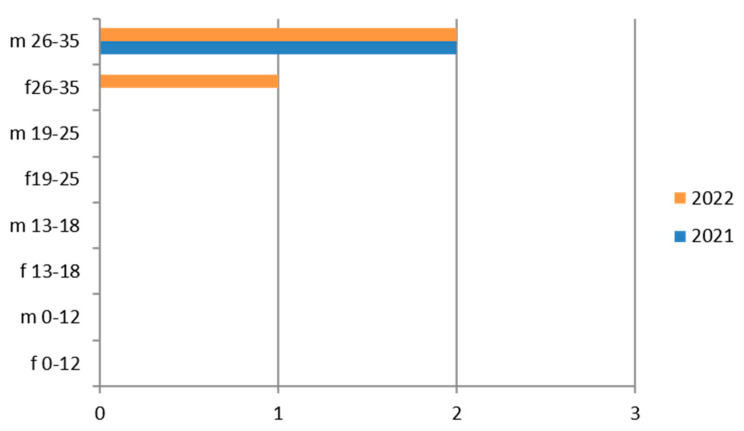
Estimated prevalence of HIV 1–2 (HIV 1–2 Ab and Ag p24), subdividing the data by sex, age group, and year (2021 and 2022).

Since the reference territory extends from the mountains to the sea, we decided to subdivide the laboratory tests in relation to the provenance as “mountain zone” (MT), “hilly zone” (HL), and “coastal zone” (CT). We also subdivided the laboratory tests by zone, sex, and age group, and we then estimated the prevalence of each microorganism in the list, subdividing the data by sex, age group, and year. We then present with graphs the representation of only the positive results of the tests carried out for each germ or type of research. In the graphs (Figure 1, Figure 2, Figure 3, Figure 4, Figure 5, Figure 6, Figure 7, Figure 8 and Figure 9), we report the data for 2022 in orange and those for 2021 in blue, while for each age group we indicate whether they are male (m) or female (f).

### 3.1. Hepatitis B

N. 8982 tests were carried out for hepatitis B surface antigen HBV (HBs Ag), with a greater predominance in the hilly area and in the female sex (Table 4). The same discussion can be conducted on the other markers of HBV infection.

Therefore, considering only the positive results, we find that the 26–35-year age group is the one with the most positive results for all HBV markers. Also in this case, there is a predominance of females (Figure 1).

The subgroup of adolescents (13–18 years) also presents positive results, particularly in the hilly area.

### 3.2. Hepatitis C

Anti-HCV IgG was tested on 9623 exams, with a predominance of the 26–35-year age group, the female sex, and the hilly area (Table 5).

Therefore, considering only the positive results, we find that the 26–35-year age group is the one with the greatest positivity, with a predominance of the male sex and the year 2021 (Figure 2).

The subgroup of adolescents (13–18 years) also presents positive results, particularly in the hilly area.

### 3.3. Chlamydia trachomatis

*Chlamydia trachomatis* was tested on vaginal swabs in 2764 exams, with a predominance in the hilly area and for the 26–35-year age group (Table 6).

Therefore, considering only the positive results, we find that the 19–25-year age group has more cases of positivity than the 26–35-year age group and their presence is greater in 2022 (Figure 3). Even the subgroup of adolescents (13–18 years) presents relatively greater positivity in the maritime area.

### 3.4. Gardnerella vaginalis

The finding of *Gardnerella vaginalis* is frequent [46] in adulthood, and its presence is linked to a change in the vaginal microbiome; it is considered an STI. In our study its presence was investigated on 998 exams, with a predominance of the hilly area and in the 26–35-year age group (Table 7).

Therefore, considering only the positive results, we find that the positivity is concentrated in the 26–35-year age group and in 2022 (Figure 4).

This germ, which is quite common in women [47], is rarely found in the adolescent group (13–18 years).

### 3.5. HPV

HPV was tested on cervical swab samples in 412 exams, showing a predominance in the hilly area and for the 26–35-year-old group (Table 8).

Therefore, considering only the positive results, we find that the group most affected is the 26–35-year age group with the greatest representation in 2022 (Figure 5).

In the group of adolescents (13–18 years old), positive cases are limited.

### 3.6. Mycoplasma/Ureaplasma

Mycoplasma/Ureaplasma was studied on vaginal cultures of 9597 exams, showing a greater presence in the hilly area and in the 26–35-year age group (Table 9).

Therefore, considering only the positive results, we find that the group most interested in positivity is the 26–35-year-old, with a clear predominance in 2022 (Figure 6).

There is a fair number of positive cases in the adolescent age group (13–18 years).

### 3.7. Neisseria gonorrhoeae

*Neisseria gonorrhoeae* was studied on 1102 cervical culture swabs, mostly in the coastal area and in the 26–35-year age group (Table 10).

Therefore, considering only the positive results, we find that the germ was isolated in only one patient in the 19–25 age group, in 2022 (Figure 7).

There are positive cases also in the adolescent age group (13–18 years).

### 3.8. Treponema pallidum

*Treponema pallidum* was investigated both with the search for TPHA/TPPA and VDRL/RPR antibodies. In the first case, 2254 exams, both male and female, were studied, while in the second examination, 2574 exams were studied (Table 11).

Therefore, considering only the positive results, we find that the Treponema Antibodies TPHA/TPPA were isolated mostly in the 26–35-year group, with a predominance in the male sex in 2022, while the 0–12 group is substantially comparable in 2022 between the two genera (Figure 8). Treponema Pallidum VDRL/RPR presents a predominance in females in 2022 (Figure 8).

There are also positive cases in the adolescent age group (13–18 years), mostly in females.

### 3.9. Virus HIV 1–2

The HIV 1–2 virus was tested on 9184 samples among males and females with a predominance in the maritime area for the female sex, while in the hilly area for the male sex. The group most interested in the investigations was the 26–35-year age group for both females and males, followed by the 19–25 group (Table 12). 

Therefore, considering only the positive results, we find that given the large number of tests carried out, the positivity is very low (Figure 9).

There are also positive cases in the adolescent age group (13–18 years), mostly in females, but not in the mountain area.

## 4. Discussion

Sexually transmitted diseases can impact quality of life [48] and even cause serious health complications. If left untreated, they can lead to neurological and cardiovascular disease, infertility, ectopic pregnancy, stillbirth, and increased risk of human immunodeficiency virus (HIV). They are also associated with stigma and domestic violence. Local epidemiological mapping is of fundamental importance for public health in the most affected areas. This could contribute to a radical decline in new sexually transmitted infections and related deaths while improving individual health, the sexual health of men and women, and universal well-being. In this way, the following objectives can be achieved: the strengthening of combined evidence-based behavioral, biomedical, and structural approaches; easier access to information about one’s sexually transmitted infection status; better access to treatment and comprehensive long-term care when needed; and the elimination of discriminatory attitudes [49].

### 4.1. Summary of Findings

The two years chosen for the study provide an interesting contrast in STI testing; during 2021, restrictions on individual freedom were in force to contain the spread of SARS-CoV-2, while the end of restrictions in 2022 allowed renewed liberty in personal behavior.

The results obtained indicate that 24,413 tests were conducted in 2021, while 96,802 were carried out in 2022 (Table 2).

The number of patients tested almost doubled from 3701 in 2021 to 5538 in 2022, in both years predominantly female patients (Table 2). In fact, considering both years studied, females seemed more careful regarding testing; almost seven times as many females as males had tests performed (7688 versus 1551).

### 4.2. Comparison with Other Studies

In the 2012 World Health Organization (WHO) report [50] about global and regional prevalence and incidence estimation of four curable STIs—chlamydia, gonorrhea, trichomoniasis, and syphilis—it was reported that among women aged 15–49 years, the estimated global prevalence of chlamydia was 4.2%, gonorrhea 0.8%, trichomoniasis 5.0%, and syphilis 0.5%; among men, the estimated chlamydia prevalence was 2.7%, gonorrhea 0.6%, trichomoniasis 0.6%, and syphilis 0.48%.

In our study, considering men and women aged 13–35 years, the estimated prevalence of HBV was 20%, HCV 21%, chlamydia 6%, Gardnerella 2%, HPV 1%, Mycoplasma/Ureaplasma 21%, Neisseria 3%, Treponema 6%, and HIV 20%.

So, the most commonly encountered pathogens were HIV 1–2, HCV, and Mycoplasma/Ureaplasma, with the highest prevalence among young people 26–35 years old, which is in line with similar results in the literature [51,52]. Taking into account data from the WHO [48], over 1 million new potentially treatable sexually transmitted diseases are contracted every day, most of which are asymptomatic. It is estimated that there are 376 million new infections each year, from one of four treatable sexually transmitted diseases (chlamydia, gonorrhea, syphilis, and trichomoniasis). Of these, trichomonas is the most common, with 156 million new cases each year, followed by chlamydia with 127 million, gonorrhea with 87 million, and syphilis with 6.3 million. The overall incidence is increasing. 

Focusing on the situations of STIs in European country, Oriol Mitjà et al. [53] performed a non-systematic review of notification data from 49 countries in the WHO European Region, covering 24 European Economic Area countries, 17 Eastern European/Central Asian countries, Switzerland, the UK, and Israel. They provide a comprehensive overview for newly diagnosed syphilis, gonorrhea, and *Chlamydia trachomatis* infections spanning a 10-year period from 2012 to 2021. One of the biggest problems remains the lack of notifications, whereby many data are underestimated [50]. 

In Europe, diagnostic tests and screening programs are very heterogeneous. It should be noted that our study reports a higher notified diagnosis of Mycoplasma/Ureaplasma in vaginal cultures, compared to national and international data [54].

Compared to WHO data, in our study, Neisseria and Treponema are much more widespread, while chlamydia has a comparable rate. This means that over time since the WHO study, the spread rate of some STIs has been increasing instead of decreasing. So, this justifies the need to continue carrying out studies like this to understand how widespread the problem is locally and therefore implement procedures to introduce corrective measures in the policies adopted, which evidently have not had great results.

### 4.3. Interpretation of the Findings

(a) Consideration about age and sex

In our study, the age group with the highest number of individuals tested was 26–35, an age which is certainly the most sexually active and, one would imagine, also more attentive to having tests performed (Table 2). Between 2021 and 2022, the number of women patients in this age group rose to 1780, a sign that the loosening of social distancing restrictions influenced individual habits. 

Though the literature data indicate that the age group 13–18 is marked by precocious sexual activity, our study data indicated that a small number of patients from this age group tested for STIs. One possible explanation is the social distancing restrictions of 2021. Of note is the different sensibility between males and females in 2022. 

Interesting data emerged for the 19–25 age group. While it would be thought that males in this group had an active sex life, the number of exams conducted for men was markedly lower than for women. Another interesting observation is that also women in this group showed greater attention to their health than the men, as already known in the literature [55]. Conversely, perhaps most of the tests for women were motivated by carelessness in their sexual behavior (for example, not insisting their partner use a condom, etc.) [56], and a consequent rush to be tested for STIs because they were aware of the risks they ran. 

Could it be hypothesized that the women in the 19–25 age group had a more active sex life than the men in this group/than the women of other age groups, marked by promiscuity and less attention to prevention than the men in this group/than the women of the other age groups?

We have no information to express ourselves regarding these possible considerations, but it is known in the literature that it is women who are more sensitive to issues relating to their health status and are more likely to turn to health facilities also for reproductive reasons [57].

The data available on STI trends in the Marche region and on specific social determinants that drive such trends is limited, if not absent.

Finally, it is possible that more women get tested than men because the former may do so even for aspecific symptoms such as vaginal discharge, while men only do so for obvious infection-related problems, for example urethritis.

(b) Consideration about geographical areas

The difference between the geographical areas is more significant towards the hill and coast, probably because the hill and coastal areas have the youngest population, so it can be assumed that the greater incidence is due to this.

The coastal areas have a significant phenomenon of female prostitution, but we have no anamnestic elements to understand whether the data on related target infections are due to this as reported in the literature (viral hepatitis, HIV, syphilis, gonorrhea, chlamydial infection, and trichomoniasis) [58,59].

Further research using socioeconomic and health data is needed to gain a greater understanding of the spread of STIs in coastal areas.

In 2022 [60], the total resident population in the mountainous zone was about 55,648, that of the hilly zone was about 153,743, and that of the coastal zone was 90,227 (taking into consideration only the municipality with the greatest population density). Thus, there were 4.3 tests/mountainous zone inhabitants, 2.5 tests/hilly zone inhabitants, and 1.8 tests/coastal zone inhabitants. Consequently, we can state that the healthcare facilities do not influence the choice to have lab tests done (Table 7).

The total number of residents aged 0–39 in the province of Macerata was 11,036, according to the Italian National Statistics Institute data. Thus, 8% of the population had STI tests done.

(c) Consideration about test results

Hepatic viruses were well represented, but sexual contact is not their only means of transmission [61]. For example, in addition to transmission by sexual contact, hepatitis B also has vertical transmission, and hepatitis C also has bloodborne transmission. In the three zones, there was not a significant increase in the number of cases of hepatic viruses during the study period. Even so, the fact that there are any new cases at all is particularly disappointing, given that the HBV vaccine has been available for years and since 1991 has been obligatory for newborns [62].

The presence of chlamydia was greater in the hilly zone, as was the case for the other microorganisms studied. Instead, the presence of *Neisseria gonorrhoeae* and syphilis (*Treponema pallidum*) increased in the coastal zone, as we had hypothesized in reference to the literature on sexual workers [63].

Looking at the data by zone, sex, and age group, it is clear that the 26–35-year age group did not present significant differences among the three zones in relation to the reference population.

It may well be that the 26–35-year age group had more STI tests conducted than the other groups because they knew about the need for STI prevention measures but nonetheless tended to engage in risky behavior (such as promiscuity and the failure to use condoms) more than the other age groups.

There was a significant presence of hepatic viruses in the test results for the 26–35-year age group. There was little difference in the presence of HBV between men and women, but there was a significant increase in HCV from 2021 to 2022 among women. However, significantly more women than men tested positive for HCV. HPV did not occur much in the 13–18 age group, probably because of Italy’s free HPV vaccination for adolescents [64].

Cases of Neisseria gonorrhea were more frequent in the coastal zone in the 26–35-year age group than in other age groups there/than in this age group in the two other zones, as were cases of syphilis (*Treponema pallidum*) and HIV, for which no tests were conducted in the mountainous zone. Of note is the evident difference in the number of males and females who had tests done for these STIs, an observation that confirms the greater consciousness among these women than among the men.

The other microorganisms studied always had greater numbers of tests conducted among the 26–35-year age group than the other groups. There was a higher frequency/number/ratio of tests per population for these tests in the hilly zone than in the other two zones.

(d) Implications of the findings

The 26–35-year age group shows a greater awareness of the importance of STI prevention, while for adolescents and young adults the results can be ambiguous. In fact, in our study, we find that the 13–18-year-old subgroup, including both males and females, represents only 3% of the sample analyzed while young adults represent 21%, but they present all the STIs analyzed in our study.

Our data on the limited spread of STIs among adolescents may seem encouraging, but the presence of any STIs in this age group is also discouraging, in the sense that it seems there is not a widespread culture of prevention in this age group and among young adults [65,66].

The present study, as already demonstrated in the literature, suggests that work is needed to improve teenagers’ access to and use of primary sexual health care services to receive useful information on sexual activities, to reinforce preventive measures in the younger age groups, and to design and implement sexual education programs.

The Italian government [67] and many associations [68,69] are promoting information campaigns among young people of the study area to give them a greater understanding of STI prevention. The literature for other countries reports the need for comprehensive, culturally sensitive, and developmentally sensitive care to address sexuality-related issues in adolescents and young adults, and this need also applies to Italy.

The authors think that the information program promoted by the Marche Region for the three-year period 2023–2025 should be aimed mainly at adolescents and young adults, preferring specific information campaigns for the male sex, who in our study appeared less present in the tested samples.

## 5. Conclusions

To contrast the phenomenon of STIs, WHO supports countries to: develop strategic plans and national guidelines; create an encouraging environment that allows people to discuss sexually transmitted diseases, adopt safer sexual practices, and seek treatment; strengthen primary prevention (availability and use of condoms, etc.); increase the integration of STI services within primary healthcare services; increase accessibility to quality, people-centered STD care; facilitate the adoption of point-of-care testing; improve and expand impactful health interventions, such as vaccination against hepatitis B and HPV and syphilis screening in priority populations; monitor and respond to antimicrobial resistance, such as in gonorrhea [48].

In this study, we provide the first report on STI prevalence in the province of Macerata. The geographical territory of the Marche region is similar in terms of social fabric and population distribution, therefore the data processed are representative of the entire region. In particular, the authors observed that sexual behavior and lifestyle habits, especially in the young population, are inevitably conditioned by the type of territory with the related services offered; therefore, in this context, the three hospitals offer a cross-section of the trend of sexually transmitted infections in a typical area of central Italy characterized by mountainous, hilly, or coastal zones. 

Data from this initial epidemiological analysis will be of great importance for the implementation of an evidence-based STI control program in the Marche region.

Prevention measures should be carried out, starting from schools or at young people’s meeting points. Finally, our investigation may lay the foundations for more extensive future studies and for the development of sexual health awareness programs. In fact, having as reference WHO recommendations and recent global studies [70,71], our small epidemiological findings call for improving the management of sexually transmitted diseases by gradually incorporating laboratory tests to support the diagnosis. STI screening strategies are essential for those at highest risk of infection, including adolescents in some settings. Therefore, health promotion campaigns should be integrated on a large scale, in addition to publishing data from peripheral realities such as the one represented in our study.

## Figures and Tables

**Table 1:** Laboratory method used.

Exam	Laboratory Method Used
Hepatitis B surface antigen HBV (HBs Ag)	Chemioluminescence
HBeAg	Chemioluminescence
Anti-HBc IgM	Chemioluminescence
Anti-HBc TOTAL (IgM + IgG)	Chemioluminescence
Anti-HBE	Chemioluminescence
Anti-HbsAG	Chemioluminescence
Anti-HCV IgG	Chemioluminescence
*Chlamydia trachomatis*—PCR on cervical swab	PCR-real time
Culture exam for Gardnerella on vaginal swab	Sowing on agar
HPV typing cervical swab	PCR-real time
Mycoplasma/Ureaplasma vaginal culture	Cultural examination on “Galleria”
*Neisseria gonorrhoeae* search culture cervical swab	Cultural sowing on biomerieux plate
*Neisseria gonorrhoeae* PCR on cervical swab	PCR real time
Treponema antibodies TPHA/TPPA	Chemioluminescence
*Treponema pallidum* VDRL/RPR	Agglutination test (manual)
Trichomonas search (PCR) vaginal swab	PCR real time or immunochromotgraphy on paper
Virus HIV 1–2 antibodies and antigens p24	Chemioluminescence

**Table 2:** Patients divided by sex and age group undergoing diagnostic tests in the years 2021–2022.

Sex	Age Group	2021	2022	Total
F	0–12	27	38	65
	13–18	60	121	181
	19–25	644	945	1589
	26–35	2223	3630	5853
	Total	2954	4734	7688
M	0–12	28	37	65
	13–18	54	44	98
	19–25	180	215	395
	26–35	485	508	993
	Total	747	804	1551
F + M		3701	5538	9239

**Table 3:** Number of separate/individual tests from 9239 patients from 2021–2022.

Sex	2021	2022	Total
F	19,642	91,300	110,942
M	4771	5502	10,273
Total	24,413	96,802	121,215

**Table 4:** Laboratory tests on hepatitis B divided into sex, age groups, according to “mountain zone” (MT), “hilly zone” (HL), and “coastal zone” (CT).

Exam	Sex	Age Group	MT	HL	CT	Total
Hepatitis B surface antigen HBV (HBs Ag)	F	0–12	14	32	16	62
		13–18	18	98	70	186
		19–25	176	582	622	1380
		26–35	576	2484	2302	5362
	M	0–12	26	42	10	78
		13–18	24	88	40	152
		19–25	68	224	248	540
		26–35	140	608	474	1222
		Total	1042	4158	3782	8982
ANTIGEN and HBV (HBeAg)	F	0–12	0	6	4	10
		13–18	2	26	4	32
		19–25	6	46	40	92
		26–35	20	84	76	180
	M	0–12	0	8	2	10
		13–18	2	8	4	14
		19–25	12	38	36	86
		26–35	32	104	94	230
		Total	74	320	260	654
ANTI-HBc Ig M	F	0–12	0	4	6	10
		13–18	2	32	4	38
		19–25	20	84	70	174
		26–35	28	144	162	334
	M	0–12	0	8	2	10
		13–18	2	14	6	22
		19–25	20	64	82	166
		26–35	34	160	160	354
		Total	106	510	492	1108
ANTI-HBc IgTotal (HBV)	F	0–12	0	8	6	14
		13–18	2	42	4	48
		19–25	56	122	94	272
		26–35	146	284	208	638
	M	0–12	0	10	4	14
		13–18	2	20	4	26
		19–25	36	136	110	282
		26–35	78	356	224	658
		Total	320	978	654	1952
ANTI-HBE HBV	F	0–12	0	8	6	14
		13–18	2	26	4	32
		19–25	2	48	44	94
		26–35	16	78	68	162
	M	0–12	0	8	2	10
		13–18	2	6	4	12
		19–25	10	44	40	94
		26–35	28	102	78	208
		Total	60	320	246	626
Anti-HbsAG (HBV)	F	0–12	16	32	14	62
		13–18	18	96	32	146
		19–25	126	328	332	786
		26–35	276	760	766	1802
	M	0–12	24	42	14	80
		13–18	22	90	28	140
		19–25	62	216	194	472
		26–35	132	518	350	1000
		Total	676	2082	1730	4488

**Table 5:** Laboratory tests on hepatitis C divided into sex, age groups, according to “mountain zone” (MT), “hilly zone” (HL), and “coastal zone” (CT).

Exam	Sex	Age Group	MT	HL	CT	Total
Anti-HCV IgG	F	0–12	4	14	14	32
		13–18	8	86	60	154
		19–25	176	592	626	1394
		26–35	696	2652	2618	5966
	M	0–12	2	21	4	27
		13–18	6	38	30	74
		19–25	70	226	236	532
		26–35	154	726	564	1444
		Total	1116	4355	4152	9623

**Table 6:** Laboratory tests on *Chlamydia trachomatis* divided into sex, age groups, according to “mountain zone” (MT), “hilly zone” (HL), and “coastal zone” (CT).

Exam	Sex	Age Group	MT	HL	CT	Total
*Chlamydia trachomatis* search (PCR) cervical swab	F	0–12		4	2	6
		13–18	15	22	25	62
		19–25	81	333	246	660
		26–35	201	1054	781	2036
		Total	297	1413	1054	2764

**Table 7:** Laboratory tests on *Gardnerella vaginalis* divided into sex, age groups, according to “mountain zone” (MT), “hilly zone” (HL), and “ coastal zone” (CT).

Exam	Sex	Age Group	MT	HL	CT	Total
Culture exam for *Gardnerella vaginalis* on vaginal swab	F	0–12	1			1
		13–18	8	11	9	28
		19–25	28	120	74	222
		26–35	74	393	280	747
		Total	111	524	363	998

**Table 8:** Laboratory tests on HPV divided into sex, age groups, according to “mountain zone” (MT), “hilly zone” (HL), and “ coastal zone” (CT).

Exam	Sex	Age Group	MT	HL	CT	Total
HPV typing cervical swab	F	13–18		5	2	7
		19–25	9	51	24	84
		26–35	19	209	90	318
		Total	28	268	116	412

**Table 9:** Laboratory tests on Mycoplasma/Ureaplasma divided into sex, age groups, according to “mountain zone” (MT), “hilly zone” (HL) and “ coastal zone” (CT).

Exam	Sex	Age Group	MT	HL	CT	Total
Mycoplasma/Ureaplasma vaginal culture	F	0–12	6	12		18
		13–18	45	75	93	213
		19–25	198	1140	837	2175
		26–35	528	3921	2742	7191
		Total	777	5148	3672	9597

**Table 10:** Laboratory tests on *Neisseria gonorrhoeae* divided into sex, age groups, according to “mountain zone” (MT), “hilly zone” (HL), and “ coastal zone” (CT).

Exam	Sex	Age Group	MT	HL	CT	Total
*Neisseria gonorrhoeae* search cervical culture swab	F	0–12		4		4
		13–18		12	30	42
		19–25	8	102	188	298
		26–35	10	234	514	758
		Total	18	352	732	1102

**Table 11:** Laboratory tests on *Treponema pallidum* divided into sex, age groups, according to “mountain zone” (MT), “hilly zone” (HL), and “coastal zone” (CT).

Exam	Sex	Age Group	MT	HL	CT	Total
Treponema antibodies TPHA/TPPA	F	0–12		4	7	11
		13–18	1	10	10	21
		19–25	36	137	141	314
		26–35	145	723	778	1646
	M	0–12		9	6	15
		13–18		7	1	8
		19–25	2	30	8	40
		26–35	17	120	62	199
		Total	201	1040	1013	2254
*Treponema pallidum* VDRL/RPR	F	0–12		4	7	11
		13–18	1	5	11	17
		19–25	41	127	196	364
		26–35	198	715	1042	1955
	M	0–12		7	8	15
		13–18	1	3	1	5
		19–25	1	17	8	26
		26–35	19	92	70	181
		Total	261	970	1343	2574

**Table 12:** Laboratory tests on HIV 1–2 divided into sex, age groups, according to “mountain zone” (MT), “hilly zone” (HL), and “ coastal zone” (CT).

Exam	Sex	Age Group	MT	HL	CT	Total
Virus HIV 1–2 antibodies and antigene p24	F	0–12		10	4	14
		13–18		76	58	134
		19–25		636	638	1274
		26–35		2816	3122	5938
	M	0–12		12	8	20
		13–18		32	12	44
		19–25		278	194	472
		26–35		750	538	1288
		Total	0	4610	4574	9184

## Data Availability

The data presented in this study are available in the laboratory archive at the Local Health Authority (AST) 3, with access via username and password. They are available upon request from Elena Bianchini.
